# Novel Decapeptides that Bind Avidly and Deliver Radioisotope to Colon Cancer Cells

**DOI:** 10.1371/journal.pone.0000964

**Published:** 2007-10-03

**Authors:** John M. Abraham, Fumiaki Sato, Yulan Cheng, Bogdan Paun, Takatsugu Kan, Alexandru Olaru, Zhe Jin, Jian Yang, Rachana Agarwal, Stefan David, James P. Hamilton, Tetsuo Ito, Yuriko Mori, Stephen J. Meltzer

**Affiliations:** 1 Department of Medicine, The Johns Hopkins University School of Medicine, Baltimore, Maryland, United States of America; 2 Sidney Kimmel Comprehensive Cancer Center, The Johns Hopkins University School of Medicine, Baltimore, Maryland, United States of America; Ordway Research Institute, United States of America

## Abstract

**Background:**

The rapidly growing field of targeted tumor therapy often utilizes an antibody, sometimes tagged with a tumor-ablating material such as radioisotope, directed against a specific molecule.

**Methodology/Principal Findings:**

This report describes the discovery of nine novel decapeptides which can be radioactively labeled, bind to, and deliver ^32^P to colon cancer cells. The decapeptides vary from one another by one to three amino acids and demonstrate vastly different binding abilities. The most avidly binding decapeptide can permanently deliver very high levels of radioisotope to the adenocarcinoma cancer cell lines at an efficiency 35 to 150 times greater than to a variety of other cell types, including cell lines derived from other types of cancer or from normal tissue.

**Conclusions/Significance:**

This experimental approach represents a new example of a strategy, termed peptide binding therapy, for the potential treatment of colorectal and other adenocarcinomas.

## Introduction

Illness and death due to colorectal and esophageal cancer constitute a monumental health care challenge in the United States and throughout the world [1], [2]. Current treatments include radiotherapy, surgery, and chemotherapy. There are a number of immunotherapies approved for use in the treatment of various types of cancers (*e.g.,* Herceptin, Rituxin, Avastin, and others) [3]–[6]. All of these immunotherapies utilize a monoclonal antibody directed against a specific cellular molecule [7], [8]. Destructive action against tumor cells is thought to involve ADCC (antibody-dependent cellular cytotoxicity), cellular lysis via the complement pathway, or the induction of apoptosis [9], [10]. Avastin is a monoclonal antibody directed against VEGF (vascular endothelial growth factor) and is approved for treatment of colorectal cancer [11]–[13].

In addition, Non-Hodgkins lymphoma (NHL) is currently treated with two approved radioimmunotherapeutic regimens: Bexxar and Zevalin. Both utilize a monoclonal antibody directed against the B-cell marker CD20 and can deliver either ^131^I (Bexxar) or ^90^Y (Zevalin) isotopes to target lymphoma cells [14], [15]. Beta-particles (electrons) generated by these isotopes can deeply penetrate cells and damage DNA, leading to cell death. However, there are currently no radioimmunotherapies approved for the treatment of patients with colorectal cancer.

The decapeptides described herein bind to and transfer isotope (^32^P) to cell lines derived from several colorectal carcinomas. Under identical experimental conditions, very little (*viz.,* less than 1% of the colon cancer cell lines' rates) of the most efficient ^32^P-labeled decapeptides bind to cell lines established from a variety of other cancers or to normal colon, kidney, or esophageal cells.

## Results

**Figure 1:**
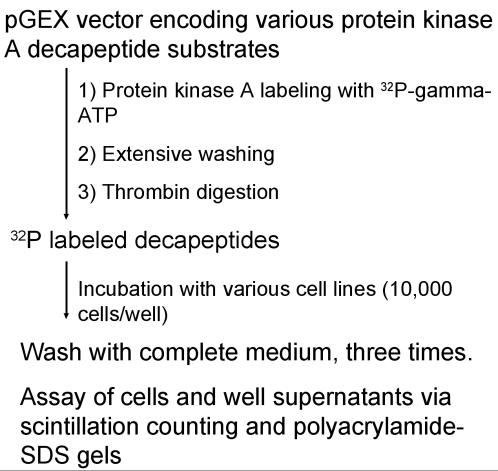
Schematic diagram of experimental approach. A bacterial recombinant expression system produced various gluthathione-S-transferase decapeptide fusion proteins which were bound to gluthatione and labeled with ^32^P utilizing protein kinase A. After washing, the labeled decapeptides were recovered after thrombin digestion and incubated at various times with several different cell lines.

We have identified nine decapeptides, differing from one another by only a few amino acids, that when labeled with ^32^P can bind to a number of colorectal carcinoma cell lines. All decapeptides contain a protein kinase A substrate sequence and are designated as MAs (Modified Adjuvant). **Figure 1** is a schematic representation of the production of the ^32^P-labeled peptides and the experimental design of assays to measure binding of peptides to cell lines.

**Figure 2:**
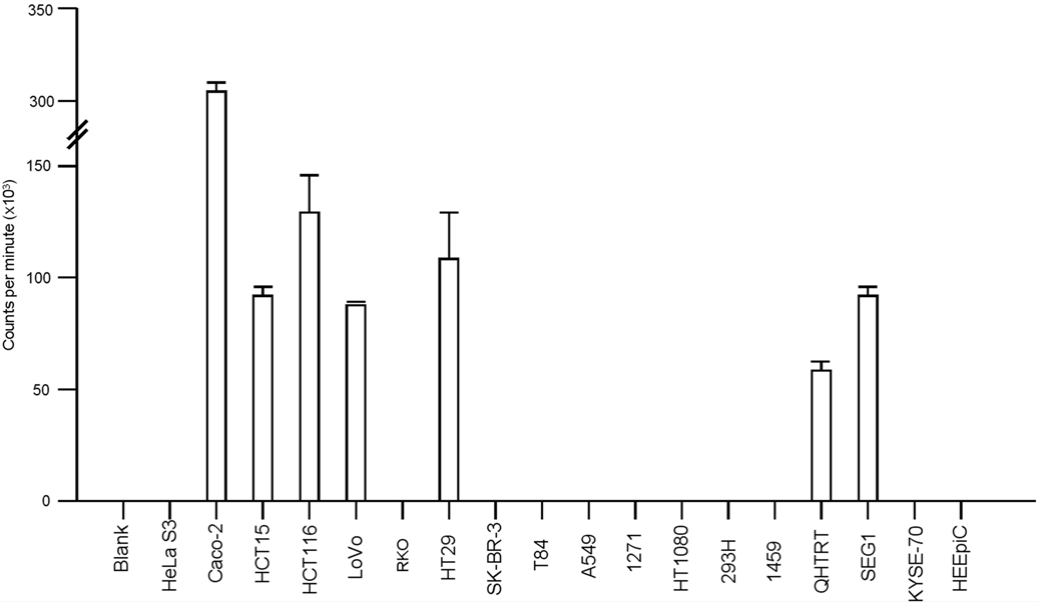
Levels of binding of decapeptide MA5 to eighteen different cell lines. The ^32^P labeled decapeptide MA5 was incubated for two hours with 10,000 cells, washed three times, and the radioactive counts of the cells determined by scintillation counting. Seven cell lines demonstrated avid binding of MA5 and are shown as bar graphs of the mean and one standard deviation in triplicate wells. The remaining eleven cell lines, along with one blank well averaged only 365 cpm. These values are so small as to not be visible at the scale used in this figure. Further information on the individual cell lines is provided in the Supplemental Information.

**Figure 2** displays the number of ^32^P counts per minute (cpm) remaining bound to eighteen different cell lines and blank wells after a two hour incubation with MA5, the most efficient binding decapeptide (see below). The Caco-2 colon adenocarcinoma cell line retained the greatest number of radioactive counts after a two-hour incubation and subsequent washes with complete medium, the average value of triplicate wells equaling 298,639 cpm per 10,000 cells. HCT116 colon adenocarcinoma cells retained an average value of 131,998 cpm per 10,000 cells. Blank wells and nonbinding cell lines had mean values of less than 550 cpm; bars representing these values are not visible at the scale used in **Figure 2**. For example, HeLa S3 cervical cancer cells only retained an average of 534 cpm per 10,000, HT1080 fibrosarcoma cells retained 367 cpm, and the human embryonic kidney cell line 293H retained 429 cpm per 10,000 cells.

Seven of the eighteen cell lines demonstrated very strong retention of radioactivity when incubated with MA5 (Modified Adjuvant radioactive peptide) with five of these being colon adenocarcinoma cell lines (Caco-2, HCT15, HCT116, LoVo, HT29), one being an esophageal adenocarcinoma cell line (SEG1), and one being a Barrett's esophagus cell line (QHTRT). In contrast, the eleven nonbinding cell lines were mostly squamous cell lines derived from carcinomas of the cervix (HeLa S3), colon (RKO), lung (1271, A549), esophagus (KYSE-70), a fibrosacroma (HT1080), or cells cultured from normal kidney (293H), colon (1459), or esophagus (HEEpiC). Nonbinding cell lines included T84, derived from a colon adenocarcinoma metastatic to lung, and SK-BR-3, isolated from a breast adenocarcinoma. The ratio of cpm retained by Caco-2 (298,639) to the average of the eleven nonbinding cell lines (365) was 818∶1. Caco-2 cells retained approximately 18% of the total radioactive counts present in the incubation well after two-hour incubation.

**Figure 3:**
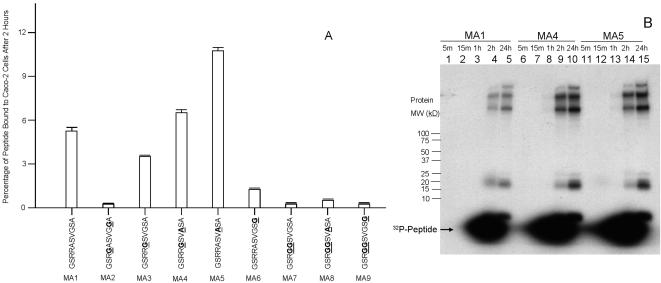
Relative levels of binding of nine ^32^P-labeled decapeptide variants. (A) Nine ^32^P-labeled different decapeptides, varying from one another by only one to three amino acids, were incubated with Caco-2 cells for two hours, the cells washed three times, and counts remaining bound to the cells are shown as a percentage of the total amount of counts for each decapeptide used. Amino acid substitutions for each variant (relative to MA1) are underlined and bolded. (B) The variants, MA1, MA4, and MA5 were incubated with Caco-2 cells for intervals varying from five minutes to two hours, washed, the adherent cells dissolved in gel loading buffer and an aliquot run on a 10%–20% gradient polyacrylamide-SDS gel. The three lanes marked “24h” (lanes 5, 10, and 15) were incubated with the respective labeled decapeptides (MA1, MA4, MA5) for two hours, washed, and the cells incubated with complete medium for 24 hours. The cells were treated as described for the other lanes of this figure.

Nine MA variants were assayed for adherence to Caco-2 cells after two hours' incubation. The relative binding level and amino acid composition of each MA variant is displayed in **Figure 3A**. Alteration of only one to three amino acids within the peptide resulted in retention differences as large as 70-fold, *e.g.,* in variant MA2 *vs.* variant MA5.

To investigate how quickly ^32^P isotope could be transferred from the peptide variants and incorporated into cellular proteins, the three most avidly binding MAs (see **Figure 3A**) were added to replicate wells containing Caco-2 cells, then washed away at varying time intervals and the cells and supernatant assayed. As shown in **Figure 3B**, substantial percentages of these ^32^P-labeled variant decapeptides bound to cells within only a few minutes, with large amounts of radiolabeled cellular proteins appearing at two hours after exposing cells to the labeled peptides. Notably, a parallel experiment in which conditions described in **Figure 3** were duplicated, but washed cells were incubated ***overnight*** in complete medium (data not shown), still revealed similar levels of ^32^P-decapeptide release and retention for all nine MAs, as described for MA5 in **Figure 2**.

**Figure 4:**
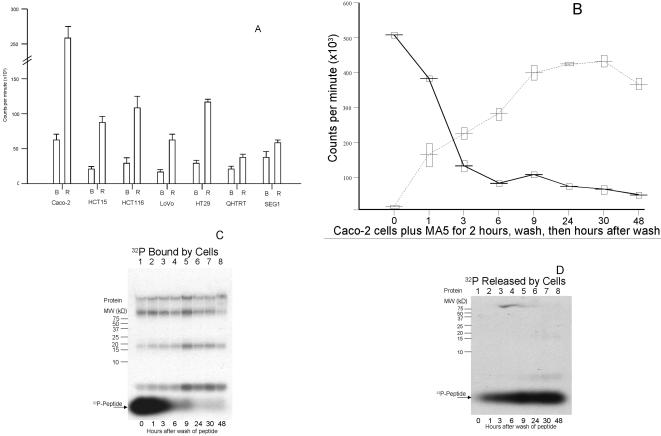
The majority of the ^32^P-labeled decapeptide MA5-bound molecules are released from Caco-2 cells. (A) The ^32^P-labeled decapeptide MA5 was incubated for two hours with seven different cell lines, the cells were washed, and complete medium was added. Following a 24 hour incubation, the number of counts per minute released into the medium (R) as well as the number of counts remaining bound to the cells (B) were determined. Each bar shows the mean and one standard deviation of triplicates wells. (B) Time course for the release and retention of the ^32^P-labeled decapeptide MA5. MA5 was incubated for two hours with Caco-2 cells, the cells washed, and the cpm released (dashed line) as well as remaining bound (solid line) to the cells determined for time intervals post-washing. Each point shows the mean plus/minus one standard deviation of triplicate determinations. C) Radioactive well contents described as bound (solid line) in Figure 4B were run on a 16.5% polyacrylamide-SDS gel and exposed to film. Immediately after washing (*i.e.,* at 0 hours), the majority of the counts were visualized as ^32^P-peptide. Over the next 48 hours, the peptide counts greatly diminished, with the majority of bound radioactivity incorporated into cellular proteins. (D) Aliquots of medium containing the released (dotted line) ^32^P-peptide MA5 were assayed at time intervals after washing, as described in Figure 4B. As time progressed, more of the ^32^P-peptide was released, reaching a plateau by 24 hours after washing.

The peptide binding, washing and assay experiment described for **Figure 2** was then repeated in the seven most avidly binding cell lines using MA5, except that after three washes of medium, 200 ul of complete medium was added to each well and the cells were incubated ***overnight*** at 37°C. **Figure 4A** shows the cpm retained by cells or released into the medium after this overnight incubation. Approximately 88% of MA5 radioactive counts initially retained by the colon cancer cell lines was released into the medium, while approximately 12% of initially retained radioactive counts were retained by cells. The two esophageal cell lines that originally retained large amounts of radioactive counts, QH-TRT and SEG1, retained 39% and 37% of their original counts, respectively, after overnight incubation. Caco-2 cells retained the greatest number of counts, averaging 58,305 cpm in triplicate wells containing 10,000 cells each. This figure represents approximately 5.8 cpm, or 348 counts per hour, per cell (*i.e.,* when extrapolated over a potential 2-week exposure period, equivalent to over 87,000 counts per cell).

**Figure 4B** shows the time course of the release of MA5 from the Caco-2 adenocarcinoma cell line over a 48-hour time period. The majority of the total counts released over the 48 hour time period are released by nine hours of incubation. **Figures 4C and 4D** consist of two autoradiograms showing the locations of the radioactive molecules described in **Figure 4B** on polyacrylamide-SDS gels. The sizes of the cellular radioactive proteins in the cells are shown in **Figure 4C**; ^32^P-labeled MA5 released into the medium is shown in **Figure 4D**. There is apparent agreement on the distribution and overall radioactivity levels in comparing **Figure 4B** and **Figures 4C and 4D**. As soon as two hours after the introduction of the radioactive peptide, a substantial portion of the isotope appears to have been transferred to higher molecular weight proteins.

## Discussion

This report describes the discovery of decapeptides that can be labeled with a high energy (1.7 Mev) beta emitter (^32^P) and can bind avidly to several different adenocarcinoma cell lines, efficiently delivering this potential tumor-ablating material to the cells. The decapeptides, termed MA for Modified Adjuvant, are protein kinase substrates. Previously, it had never been shown or suspected that this substrate, when labeled with a tumor-ablating material such as ^32^P, could bind to and transfer the radioisotope to a cell line after one to two hours of incubation. Moreover, we have shown for the first time that transfer of isotope from these decapeptides is restricted to cell types derived from primary colon and esophageal adenocarcinomas. For example, exposure of certain colon cancer cell lines *(e.g*. Caco-2) to the most avidly binding labeled peptide, MA5, for a two-hour period resulted in the transfer of a radioactive dose of over 29 counts per minute per cell after a two hour incubation, wash, and immediate determination of the retained radioactivity.

The incubation of ^32^P-labeled decapeptide with certain cell lines resulted in large amounts of peptide being retained after a two-hour incubation, but a substantial proportion of this bound peptide was released after an overnight incubation. For example, after incubation of the labeled variant MA5 with Caco2 cells for two hours, three wash steps, and overnight incubation in medium, 88% of the originally retained ^32^P isotope was released. However, the 12% that was retained by cells still represented 5.8 cpm per cell, extrapolating to over 8,300 counts per cell per day. In addition, radioactivity that was still retained by cells after overnight medium incubation was permanently incorporated into a variety of cellular proteins, as demonstrated by polyacrylamide gel electrophoresis of post-exposure cellular lysates

Among 18 cell lines assayed for their ability to bind the decapeptides, seven demonstrated very high retention of isotope after two-hour incubation. Although all seven of these lines released from 63% to 88% of this radioactivity after an overnight incubation, the amount of isotope that was retained overnight was still substantial. Of these seven cell lines, five were derived from colorectal adenocarcinomas, one from an esophageal adenocarcinoma, and one from a Barrett's metaplasia specimen. The 11 cell lines that did not bind the radioactively labeled decapeptide MA5 were derived from a variety of tissue origins. These included squamous cell carcinomas of the cervix, lung, breast, and a fibrosarcoma, as well as normal kidney, colon, and esophageal tissues.

The majority of approved immunotherapeutic regimens for cancer involve an antibody directed against a specific cellular molecule [16]. These agents can function by binding to the cell surface and may utilize ADCC, complement activation, or cellular apoptosis. The antibodies may also be coupled to a tumor-ablating agent, such as toxins or radioisotopes [17]-[21]. The addition of isotope to peptides, and their use for both diagnostic and therapeutic purposes, is an active area of biomedical research [22]-[25]. Our work utilizes protein kinase A substrates labeled with ^32^P isotope. A high-energy beta-emitting radioisotope results in an electron pathlength range of up to 5 mm, permitting substantial penetration of solid tumors. Due to a predicted “bystander” effect, one beta particle will penetrate hundreds or thousands of cells within the tumor, even those not directly binding the decapeptide. Moreover, since the molecular weights of these minuscule decapeptides proteins are far lower than the exclusionary molecular weight limit of the filtering kidneys, these peptides should be rapidly eliminated in the urine, leading to reduced systemic toxicity. Thus, it should be feasible for both a radioactive dose and unbound radioactivity to be eliminated easily and in a relatively short period of time. We anticipate that additional known enzyme substrates may eventually be identified as potential vehicles for the specific delivery of anti-tumor agents to cancer cells and that potential cancer therapeutic regimens employing this peptide or other similar substances might be the newest strategy for peptide binding therapy.

## Materials and Methods

Production of the ^32^P-labeled decapeptides: Different DNA oligomers were cloned into pGEX-4T-1 (GE Healthcare) which yield various decapeptides after thrombin cleavage designated MA1 through MA9 (Modified Adjuvant). The protein sequences are: MA1, GSRRASVGSA; MA2, GSRGASVGGA; MA3, GSRRGSVGSA; MA4, GSRRGSVASA; MA5, GSRRASVASA; MA6, GSRRASVGSG; MA7, GSRGGSVGSA; MA8, GSRGGSVASA; MA9, GSRGGSVGSG. DH5-α bacteria containing these clones were grown overnight in LB (containing 100 µg/ml ampicillin), diluted 1/10 in LB-Amp and grown at 37°C for two hours. IPTG was added to 1 mM and the culture grown at 37°C for five hours. Ten ml of each culture were centrifuged and the cell pellet resuspended in 1 X TBS containing 100 µg/ml lysozyme. After two cycles of freeze-thaw, the lysate was centrifuged and the supernatant was mixed with 100 µl of Sepharose-Glutathione for two hours at RT. Each pellet was washed three times with 1 X TBS, and the bound recombinant fusion proteins were labeled with ^32^P using protein kinase A and ^32^P-γ-ATP according to the manufacturer's instructions (Sigma, St. Louis, Mo.). The pellet was washed four times with 1 X PBS and the labeled decapeptide was cleaved and released into the supernatant with thrombin (GE Healthcare).

Assay of the binding of ^32^P-labeled decapeptides to cell lines: Cell lines were grown in complete medium containing 10% bovine fetal serum (heat inactivated). In each well of a 96-well plate, 10,000 cells from various cell lines were grown overnight in complete medium. Ten µl of the labeled-peptide in 1 X PBS and 90 µl of complete medium were added to each well and incubated at 37°C at various times of up to two hours. The peptide-medium was removed and one µl added to 100 ul gel loading buffer and counted by scintillation counting for the probe control or run on a polyacrylamide-SDS gel (Biorad).The adherent cells were briefly and gently washed with complete medium three times and some wells were assayed immediately by adding 100 µl of gel loading buffer to each well and run on a gel or counted in a scintillation counter. Other wells had 100 µl complete medium added and incubated for a further time period. Samples were either counted in a liquid scintillation counter or run on polyacrylamide-SDS gels, exposed to x-ray film, and the film developed.

## References

[1] Edwards BK, Brown ML, Wingo PA, Howe HL, Ward F (2005). Annual report to the nation on the status of cancer, 1975-2002, featuring population-based trends in cancer treatment.. J. Natl. Cancer Inst..

[2] Jemal A, Siegel R, Ward E, Murray T, Xu J (2007). Cancer Statistics, 2007.. CA Cancer J. Clin..

[3] Slamon DJ, Leyland-Jones B, Shak S, Fuchs H, Paton V (2001). Use of Chemotherapy plus a monoclonal antibody against HER2 for metastastatic breast cancer that overexpresses HER2.. N. Engl. J. Med..

[4] Romond EH, Perez EA, Bryant J, Suman VJ, Geyer CE (2005). Trastuzumab plus adjuvant chemotherapy for operable HER2 positive breast cancer.. N. Engl. J. Med..

[5] Tan-Chiu E, Yothers G, Romond E, Geyer CE, Ewer M (2005). Assessment of cardiac dysfunstion in a randomized trial comparing doxorubicin and cyclophosphamide followed by placlitaxel, with or without Trastuzumab as adjuvant therapy in node positive, human epidermal growth factor receptor 2-overexpressing breast cancer.. J.Clin. Oncol..

[6] Davis TA, Grillo-Lopez AJ, White CA, McLaughlin P, Czuczman MS (2000). Rituximab anti-CD20 monoclonal antibody therapy in non-Hodgkin's lymphoma: safety and efficacy of re-treatment.. J. Clin. Oncol..

[7] Nahta R, Esteva FJ (2007). Trastuzumab:triumphs and tribulations.. Oncogene.

[8] Presta LG, Chen H, O'Connor SJ, Chisholm V, Meng YG (1997). Humanization of an anti-vascular endothelial growth factor monoclonal antibody for the therapy of solid tumors and other disorders.. Cancer Res..

[9] Clynes RA, Towers TL, Presta LG, Ravetch JV (2000). Inhibitory Fc receptors modulate in vivo cytoxicity against tumor targets.. Nat. Med..

[10] Siberil S, Dutertre CA, Fridman WH, Teillaud JL (2007). FcgammaR: The key to optimize therapeutic antibodies?. Crit. Rev. Oncol. Hematol..

[11] Rini BI, Rathmell WK (2007). Biological aspects and binding strategies of vascular endothelial growth factor in renal cell carcinoma.. Clin. Cancer Res..

[12] Van Cutsem E, Peeters M, Siena S, Humblet V, Hendlisz A (2007). Open-label phase III trial of panitumumab plus best supportive care compared with best supportive care alone in patients with chemotherapy-refractory metastatic colorectal cancer.. J. Clin. Oncol..

[13] Cilley JC, Barfi K, Benson AB, Mulcahy MF (2007). Bevacizumab in the treatment of colorectal cancer.. Expert Opin. Biol. Ther..

[14] Wiseman GA, White CA, Sparks RB, Erwin WD, Podoloff DA (2001). Biodistribution and dosimetry results from a phase III prospectively randomized controlled trial of Zevalin radioimmunotherapy for low-grade, follicular, or transformed B-cell non-Hodgkin's lymphoma.. Crit. Rev. Oncol. Hematol..

[15] Vose JM (2004). Bexxar: novel radioimmunotherapy for the treatment of low-grade and transformed low-graade non-Hodgkin's lymphoma. (2004). Oncologist.

[16] Zafir-Lavie I, Michaeli Y, Reiter Y (2007). Novel antibodies as anticancer agents.. Oncogene.

[17] Kreitman RJ (2006). Immunotoxins for targeted cancer therapy.. *AAPS J*..

[18] Kreitman RJ, Pastan I (2006). Immunotoxins in the treatment of hematologic malignancies.. Curr. Drug Targets.

[19] Kreitman RJ (2003). Recombinant toxins for the treatment of cancer.. Curr. Opin. Mol. Ther..

[20] Boerman OC, Koppe MJ, Postema EJ, Corstens FH, Oyen WJ (2007). Radionuclide therapy of cancer with radiolabeled antibodies.. Anticancer Agents Med. Chem..

[21] Wu AM, Senter PD (2005). Arming antibodies: prospects and challenges for immunoconjugates.. Nat. Biotech..

[22] Okarvi SM (2004). Peptide-based radiopharmaceuticals: Future tools for diagnostic imaging of cancers and other diseases.. Med. Res. Rev..

[23] Aina OH, Sroka TC, Chen ML, Lam KS (2002). Therapeutic Cancer Targeting Peptides.. Biopolymers.

[24] Wangler C, Buchmann I, Eisenhut M, Haberkorn U, Mier W (2007). Radiolabeled peptides and proteins in cancer therapy.. Protein Pept. Lett..

[25] Aina OH, Marik J, Liu R, Lau DH, Lam KS (2005). Identification of novel targeting peptides for human ovarian cancer cells using “one-bead one-compound” combinatorial libraries.. Mol. Cancer Ther..

